# Cortisol Awakening Response, Self-Reported Affect and Exam Performance in Female Students

**DOI:** 10.1007/s10484-019-09449-9

**Published:** 2019-09-05

**Authors:** Wladyslaw Losiak, Julia Losiak-Pilch

**Affiliations:** 1 Institute of Psychology, Jagiellonian University, ul. Ingardena 6, 30-060 Kraków, Poland; 2 Institute of Pedagogy, Jagiellonian University, ul. Batorego 12, 31-135 Kraków, Poland

**Keywords:** Cortisol, Cortisol awakening response, Affect, Performance, Exam stress, Students

## Abstract

The purpose of the study was to find whether there were differences in cortisol awakening response (CAR) between a neutral day and an exam day in a group of female students and to explore possible relationships between CAR, self-reported affect, and exam performance. A group of 25 female students took samples of their saliva using Salivettes at the moment of waking and after 30 min. They then described their affect using the PANAS scale. Measures were taken twice: three days before an examination and on the day of the examination. The level of free cortisol in saliva samples was determined using enzyme immunoassay. The integrated volume of cortisol (CARauc) was significantly higher on the day of the exam than on the neutral day. There were also significant differences in affect, with negative higher and positive lower on the exam day, but correlations between cortisol measures and self-reported affect were low and not significant. A negative relationship between integrated volume of cortisol (CARauc) and exam performance was also found. Anticipated exam stress caused a significant increase in CAR in female study participants when compared to a neutral day, but only in the case of integrated volume of cortisol over the waking period (CARauc). The negative relationship between this measure and exam performance can be explained by attributing CARauc to negative expectations concerning the anticipated exam.

## Introduction

Cortisol awakening response (CAR) indicating an increase in cortisol level after waking as a part of the diurnal circle of cortisol secretion is a variable used in many studies as it has been found to be related to important psychosocial and mental health characteristics, including life stress (Chida and Steptoe 2009). While studies on CAR correlates are not unambiguous, some have shown that increased CAR is related to job stress (Alderling et al. 2006; Chida and Steptoe 2009), perceived chronic stress (Wust et al. 2000), and perceived loneliness (Steptoe et al. 2004). On the other hand, negative correlations have been found between CAR and symptoms of PTSD or burnout (Chida and Steptoe, 2009). Differences in CAR have also been found in the same subjects when cortisol rise was compared between weekdays and weekends, when CAR was generally lower (Clow et al. 2004). This effect was attributed to perceived work overload and chronic worrying on weekdays (Schlotz et al. 2004). Generally speaking studies indicate that while flat CAR is observed in cases of PTSD or chronic fatigue, in other cases increased CAR is related to anticipation of potentially stressful events of the day in the majority of healthy individuals (Clow et al. 2004).

There is also evidence that increased CAR is observed in patients suffering from somatic diseases such as coronary artery disease (Btattacharyya et al. 2008), upper respiratory illness (Edwards et al. 2003), and obesity (Wallerius et al. 2003). Increased CAR has also been shown to be associated with psychopathology such as bipolar illness (Ellenbogen et al. 2004), and borderline personality disorder (Lieb et al. 2004).

Some differences were found between two measures of CAR: a cortisol increase following waking (CARi) and integrated volume of cortisol over the waking period (CARauc). These measures result from two formulas for computation of morning increase of cortisol level, namely area under the curve with respect to the ground (CARauc) and area under the curve with respect to increase (CARi) (Pruessner et al. 2003). Moreover, it is recommended to employ both formulas when analysing data sets with repeated measures (Pruessner et al. 2003).

In psychotherapy, CARauc was higher in patients with diagnosed depression than in a control group, but CARi was reduced (Huber et al. 2006). CARi was positively related to job stress and general life stress but was negatively related to fatigue or burnout; however, CARauc was positively related to general life stress but negatively to PTSD symptoms (Chida and Steptoe 2009). Generally speaking changes observed in morning cortisol secretion in clinical groups indicate dysregulation of endocrinological processes in psychopathology.

While the question of the possible function of CAR remains open, it is hypothesized that its main role is to mobilize energy during the transition from sleep to wakefulness, to prepare the organism for the expected demands of the day, and to facilitate orientation of the self in time and space (Kudielka and Wust 2010). If we assume that the purpose of cortisol secretion is to enhance daily activities, then the issue of the cortisol performance relationship arises. Empirical results have been rather inconclusive so far. According to some recent studies, cortisol level has been found to be positively related to prospective memory function (Glienke and Piefke 2016), visuospatial memory (Human et al. 2013), or delayed retrieval of educationally relevant material (Hupbach and Fieman 2012). However, in the case of the latter study the effect was observed only in male participants. At the same time, it has been found to be negatively related to working memory tasks in healthy young men (Schoofs and Wolf 2009), explicit memory and cognitive performance in both male and female students (Kazen et al. 2012), cognitive performance in business executives (Teixeira et al. 2015), conditions of simulated firefighting in unexperienced volunteers (Robinson et al. 2013), and working memory performance in police officers exposed to an uncontrollable situation with the risk of being shot (Taverniers et al. 2011). It is worth noting that in the latter study a positive effect of stress-induced cortisol on perceivably acquired task-related skills and competencies was observed. Moreover, other studies showed no effect of cortisol on performance in medical students (Singh et al. 2012), while the results from a rather small sample of athletes showed no relationship between CAR and performance in sport (Diaz et al. 2013).

The purpose of the present study was to find whether there were differences in cortisol awakening response between a neutral day and an exam day in a group of female students and to explore possible relationships between CAR, self-reported affect, and exam performance. Considering the results of many studies on factors influencing CAR (see Clow et al. 2004) we formulated the hypothesis that CAR on an exam day would be higher than on a neutral day. Moreover we wanted to show that two measures of cortisol (at the awakening and 30 min later) are sufficient for estimating CAR as an indicator of stress.

In terms of the relationship between CAR and exam performance, we did not formulate any hypothesis since the results of the relationship between cortisol secretion and performance were rather inconclusive. In addition, cortisol was usually measured in a close time relationship to performance; however, in the present study it was measured in the morning and the exam took place in the afternoon. Exploration of the possible relationship between CAR and exam performance was based on the assumption that CAR reflected anticipated exam stress and thus could be related to exam performance.

## Methods

### Participants and Procedure

Twenty-five female first year students, aged 19–21 were recruited during lectures. Only participants who declared that they were neither taking medication nor being treated for endocrine disorders were recruited. They were asked to participate in a study concerning affective reactions during an exam session that would require completing questionnaires and taking saliva samples twice on two different days. They all agreed to participate and gave informed consent. They were then given detailed written and oral instructions describing the procedure.

The first measurement took place 3 days before the exam. Following the written instructions, participants noted the time and collected the first saliva sample using a Salivette in the morning immediately after waking. They also reported waking time and sleep duration. They were asked not to drink coffee or tea, eat, engage in physical exercise, or brush their teeth for a period of 30 min, after which they collected the second saliva sample and completed the PANAS scale to measure affect. They put both samples in a freezer until they were collected by experimenters. The saliva sampling procedure was designed according to suggestions by Adam and Kumari (2009) concerning methods to optimise compliance with saliva collection protocols.

The second measurement took place in the morning on the day of the exam according to the procedure described above. The exam took place in the afternoon at 3 PM. The exam could be considered as “high stakes”—it was the final exam after one semester course during first year of studies. Failing an exam could result in being not allowed to continue studies. The sleep duration on both measurement days was similar and the difference was not significant (dependent samples t test t(25) = 1.069, p = 0.295, neutral day: Mean 411 min, SD 39 min, exam day: Mean 407 min, SD 34 min).

The study protocol, information on the study, informed consent, and related materials were submitted and approved by the ethics committee of Institute of Psychology, Jagiellonian University in Cracow. Informed consent was obtained from all participants in the study.

### Self-Report Measures

The Positive and Negative Affect Schedule (PANAS) is a 20-item adjective measure that assesses two dimensions of affect (Watson et al. 1988) with a Positive and a Negative Affect scale. Participants are asked to rate the extent to which they experienced each particular emotion within a specified period using a 5-point Likert scale, with values ranging from “very slightly” to “extremely”. Participants in the current study were asked to describe how they felt when they completed the scale, about 30 min after waking, and right after the second saliva sample was collected.

### Salivary Cortisol Measures

Free salivary cortisol levels were used as an indicator of cortisol secretion after waking. Saliva samples were collected by a standard procedure using a Salivette (Sarstedt, Numbrecht, Germany). The participants were asked to place a cotton wad in their mouth, chew it for approximately 45 s to induce salivation, and then transfer it to a sterile tube. The labelled tubes were stored in a freezer (at − 20 °C) before being sent to a specialized laboratory. There, the samples were thawed and centrifuged before free cortisol level was determined in duplicate by enzyme immunoassay with a commercially available kit (ELISA—Demeditec, Germany). The intra-assay coefficients of variance were less than 10%.

## Results

Descriptive statistics for salivary cortisol (nmol) are given in Table 1 together with reference values reported by Clow et al. (2004), showing mean values of cortisol volume in healthy adult participants at waking and after 30 min in neutral conditions. Values on a neutral day obtained in this study are similar; however, much greater interindividual variance of cortisol volume 30 min after waking on the day of the exam should also be noted.

**Table 1:** Mean values of salivary cortisol (nmol) on waking and after 30 min on a neutral and an exam day in the studied sample of female students

	Neutral day	Exam day	Normative data of healthy adults (Clow et al. 2004)
	Mean (SD)	Mean (SD)	Mean (SD)
Waking	11.74 (6.56)	18.90 (7.58)	11.6 (4.6)
+ 30 min	18.88 (9.23)	28.04 (20.31)	20.0 (5.9)

In further analyses, we transformed logarithmically cortisol nmol values to obtain normal distribution and no outliers were observed. Descriptive statistics and correlations of variables used in the analyses are given in Table 2. Two measures of CAR were calculated according to formulas by Pruessner et al. (2003): the first represents integrated volume of cortisol over the waking period (CARauc); the second represents increase of cortisol following waking (CARi).

**Table 2:** Descriptive statistics and intercorrelations of variables in the study

	Mean (SD)	CARauc N	CARi N	Negative affect N	Positive affect N	CARauc E	CARi E	Negative affect E	Positve affect E
CARauc N	1.11 (.25)								
CARi N	0.18 (.11)	− .30							
Negative affect N	15.54 (5.77)	− .14	.11						
Positive affect N	23.69 (7.68)	.03	.14	− .20					
CARauc E	1.29 (.28)	.57**	− .19	− .10	− .20				
CARi E	.08 (.09)	.03	− .04	.07	− .13	− .15			
Negative affect E	20.38 (7.26)	.11	− .15	.65**	.07	.09	− .16		
Positive affect E	21.23 (7.24)	− .35	.43*	.00	.69**	− .26	− .28	− .01	
Exam score	41.76 (8.08)	− .09	− .22	.19	.30-	− .45*	.06	.12	.25

CAR values logarithmically transformed

*N* neutral day, *E* exam day

*p < .05, **p < .01

We used ANOVA for repeated measures to test differences in CAR on a neutral and exam day. There was no significant difference in the increase of cortisol following waking (CARi - F (1,24) = 2.091, p = .16, eta-squared = 0.080) between the 2 days, but there was a significant difference in integrated volume of cortisol over the waking period (CARauc – F (1,24) = 13.788, p = .001, eta-squared = 0.365). The integrated volume of cortisol was higher on the day of the exam than on the neutral day. Moreover, we found significant difference in a single measure of cortisol namely cortisol level measured 30 min after awakening which was higher on the exam day compared to neutral day (F (1,24) = 7.644, p = .01, eta squared = 0.242).

We performed an analogous ANOVA on the measures of self-reported negative and positive affect. Negative affect was significantly higher in the morning on the day of the exam when compared with the neutral day (F (1,24) = 19.486, p = .0001, eta-squared = 0.438) and the positive affect was significantly lower on the day of the exam (F (1,24) = 4.556, p = .04, eta-squared = 0.154).

We used hierarchical regression analysis to evaluate the effect of the CAR and self-reported affect in the morning of the exam day on exam performance, as measured by the number of correct answers in the test (Table 3). Only one variable—the integrated volume of cortisol over the waking period (CARauc)—on the examination day was a significant predictor of exam performance. The relationship was negative: the higher the CARauc on the day of the exam, the lower the exam performance. Neither the increase of cortisol following waking (CARi) nor negative or positive self-reported affect on the day of the exam contributed to a significant increase of explained variance in the regression. It should also be noted that the amount of explained variance was generally low. Similar relationships were shown in the regression analysis in which a single measure of cortisol measured 30 min post awakening on the day of the exam was used instead of CAR measures (Table 4).

**Table 3:** Exam performance (number of correct answers) regressed on CAR on exam day (CARauc and CARi) and self-reported affect (negative and positive): results of hierarchical regression

Variables	Beta	R2	R2 change	F (model)	df
CARauc E	− .446*	.19	,19*	5.476*	1, 22
CARauc E CARi E	− .451* − .026	.20	.001	2.624	2, 21
CARauc E CARi E Negative affect E	− .502* − .009 .272	.27	.071	2.477	3, 20
CARauc E CARi E Negative affect E Positive affect E	− .446* .047 .258 .141	.28	.015	1.90	4, 19

*E* exam day

*p < .05

**Table 4:** Exam performance (number of correct answers) regressed on cortisol level measured 30 min post awakening on exam day and self-reported affect (negative and positive): results of hierarchical regression

Variables	Beta	R2	R2 change	F (model)	df
Cortisol + 30 E	− .431*	.19	.19*	5.015*	1, 22
Cortisol + 30 E Negative affect E	− .474* .253	.25	.06	3.459*	2, 21
Cortisol + 30 E Negative affect E Positive affect E	− .438* .242 .080	.253	.005	2.258	3, 20

*E* exam day

*p < .05

## Discussion

This study demonstrates that a simplified approach to assessing the CAR is relevant to anticipated exam stress. That is, we found a significant increase in integrated volume of cortisol over the waking period (CARauc) on an exam day when compared to a neutral day. Increase of cortisol following waking (CARi) did not change significantly between the two measurement days. While expectations that CAR would be sensitive to anticipation of potentially stressful events during the day were generally confirmed, it should be noted that the effect was not observed in the second measure of CAR: increase of cortisol following waking (CARi). This result is somewhat confusing since there is a growing conviction that the actual indicator of CAR is the increase of cortisol following waking (CARi) (Stalder et al. 2016), not the integrated volume (CARauc). However, the difference in CARauc between the neutral and the exam day was quite pronounced in this study; thus, it can be attributed to participants’ anticipation of stress.

Since CARauc is considered to be a more robust measure of morning cortisol increase and according to obtained results it was higher on the exam day thus indicating anticipated stress, it seems that this measure could be used in smaller clinical applications when lab resources are more limited. It is also worth noting that results of our study indicate that two sample approach to measure CAR, which is usually considered as weak, is in fact sufficient to estimate CARauc as an indicator of stress. Moreover, it seems possible that even only one sample—cortisol measured 30 min after awakening—can be treated as an indicator of stress. Obviously not at the individual level due to differences in cortisol secretion but only when analysing group differences.

When self-reported morning affect was considered, the effect was predictable: negative affect was higher and positive affect was lower on the day of the exam, compared to the neutral day. Observed changes in affect can be attributed to anticipation of a potentially stressful event during the day. However, it should be noted that correlations between self-reported affect and cortisol measures were not significant and rather low.

CARauc—one of the measures of CAR—was a significant negative predictor of exam performance, while neither CARi nor self-reported affect were related to performance. Although this result should be treated as tentative, it suggests that anticipated stress indicated by integrated volume of cortisol over the waking period is related to impaired performance on a mental task during the day. In a similar way cortisol level measured 30 min after awakening showed significant relationship with exam performance. Considering the period between morning cortisol sampling and early afternoon exam performance, the observed effect should not be explained in terms of cortisol influence, as it was in studies in which cortisol was measured in direct time relationship to performance. It is more likely that participants who felt less self-confident and were aware of being insufficiently prepared for the exam anticipated the exam as potentially more stressful and this was reflected in CAR on the exam day. Future studies should include measures of time studying for exam and the participants confidence about the exam.

Generally speaking results suggest that greater than usual cortisol increase after awakening can be treated as the physiological indicator of anticipated stress. Moreover this measure is free from individual biases that influence self-reported ratings of stress or affect. Low correlations between CAR and self- reported affect, observed in the reported study, can be attributed to such biases influencing personal estimations of experienced affective states.

Some limitations of the study should also be noted. The process of the collection of saliva samples was not controlled and participants’ compliance with the sampling procedure could have been unsatisfactory in some cases. Moreover, the two-sample protocol of morning cortisol collection is considered rather weak and usually three or more collections are recommended (Stalder et al. 2016). Having said that it should also be noted that peak levels are typically observed 30 min post awakening (Clow et al. 2004). Moreover, significant differences in CARauc obtained in our study seem to indicate that two sample approach can be sufficient. While the aforementioned issues could contribute to measurement error of cortisol levels, in general they do not explain the differences in observed effects of CARauc and CARi since both indicators of CAR were based on the same measures. It should also be noted that the obtained results relate only to female participants, but some gender differences in cortisol reactivity have previously been reported (Clow et al. 2004).
